# Night Watch: A Survey of Older Adult Sleep-Tracking

**DOI:** 10.3390/bs16060917

**Published:** 2026-06-03

**Authors:** Thomas James Hoffmann, Lisa Gualtieri

**Affiliations:** Cedars-Sinai Medical Center, Los Angeles, CA 90048, USA

**Keywords:** sleep, trackers, wearables, older adults, behavior change

## Abstract

Consumer sleep-tracking wearables are increasingly used by older adults, yet their impact on behavior is underexamined. This study examined how U.S. adults 50 years of age and older use and interpret sleep-tracking data from Fitbit, Apple Watch, and Oura Ring, and how users perceive its influence on sleep-related routines and behaviors. A convergent parallel mixed-methods design was used, combining Likert-scale items and open-ended responses from a survey of older adults with one month or more of wearable use. Quantitative findings (*n* = 103) indicated high perceived usability (M = 4.14) and usefulness (M = 4.27). The behavior-change composite score (M = 3.78, SD = 0.87) was significantly greater than the neutral midpoint (*p* < 0.001), suggesting perceived improvements in sleep-related routines. Qualitative analysis (*n* = 86) identified four themes: sleep tracking as a reflective feedback system, incremental routine adjustments, selective interpretation of metrics, and desire for more actionable guidance. Overall, sleep-tracking devices were associated with increased awareness and modest behavioral adjustments rather than substantial change. These findings suggest that wearables function primarily as reflective tools supporting gradual habit refinement. Future research should evaluate whether these incremental changes translate into sustained improvements in sleep and daytime functioning.

## 1. Introduction

Sleep changes are a well-documented phenomenon of aging, characterized by increased sleep fragmentation, reduced sleep continuity, and night-to-night variability. These alterations are clinically meaningful, given that insufficient or disrupted sleep in later life has been associated with cognitive decline, cardiometabolic disease, mood disturbance, and reduced functional independence (Liu et al., 2025). As the desire to “age in place”—a concept consistently expressed as important to older adults—grows (Lampkin, 2024a; Sharma et al., 2021), scalable and unobtrusive approaches to tracking sleep patterns over time have become increasingly important (Liu et al., 2025; Sharma et al., 2021).

To support independence and health monitoring, many older adults are incorporating personal health technologies into their daily routines. Approximately one-third of Americans 50 years of age or older report owning a wearable device (Kakulla, 2024). In the general population, widely used wrist-worn and ring-based wearables, including Fitbit, Apple Watch, and Oura Ring, offer sleep-tracking features alongside other health metrics, such as physical activity, cardiovascular, and respiratory indicators (Robbins et al., 2024). These devices have been shown to reliably estimate certain sleep parameters in healthy older individuals while offering low-cost, unobtrusive insight into normal, age-related changes in sleep patterns (Robbins et al., 2024; de Zambotti et al., 2019). By supporting longitudinal monitoring under free-living conditions, they provide ecologically valid sleep data that may be particularly relevant for aging populations, whose sleep is often influenced by environmental, behavioral, and health-related factors (Ghazi et al., 2025).

Despite widespread adoption, important questions remain regarding the performance and real-world impact of consumer sleep-tracking wearables. Evidence suggests that the accuracy of these devices is reduced among older age groups, with validation studies demonstrating age-related declines in agreement between wearable-derived estimates and polysomnography (PSG) (Searles et al., 2026). Beyond technical performance, qualitative research suggests that sustained use among older adults depends on whether device feedback is meaningful, interpretable, and aligned with personal health goals. Older users tend to view wearables as practical health tools rather than novelty technologies, and when sleep metrics appear conflicting or implausible, trust may erode, anxiety may increase, and engagement may decline (Moore et al., 2021).

This study examines how adults aged 50 years and older experience, interpret, and integrate sleep-tracking wearables into everyday life, focusing on bedtime and morning routines. Technical limitations of these devices are well documented, but their real-world behavioral impact remains underexamined. Current attitudes remain mixed, with ongoing questions regarding usefulness and behavioral adaptations (Kakulla et al., 2025). Perceived positive effects are generally noted by users (Project Catalyst & HomeLab, 2016); however, sleep-tracking rarely results in sustained improvements in sleep hygiene and has been shown to strengthen the link between short sleep and anxiety (Dion et al., 2025).

The primary aim of this study was to examine how adults aged 50 years and older who use, or have previously used, a sleep-tracking wearable device for at least one month perceive, interpret, and integrate sleep-tracking data into their bedtime and morning routines, with particular focus on perceptions of device usability, usefulness, and behavioral influence. The secondary aim was to explore whether perceived behavioral influence varies across demographic and contextual factors, including age, gender, race and ethnicity, education level, health status, device type, length of use, and method of acquisition. The central research question guiding this work was: How do adults aged 50 years and older experience, interpret, and incorporate data from sleep-tracking wearables into their bedtime and morning routines? It was hypothesized that adults aged 50 years and older who use, or have previously used, sleep-tracking wearables for at least one month would report perceived positive influences of sleep-tracking data on their sleep-related routines and behaviors. A second hypothesis proposed that these routines would be associated with demographic and contextual factors, including age, gender, race and ethnicity, education level, health status, device type, length of use, and method of acquisition.

## 2. Methods

### 2.1. Study Design

A cross-sectional convergent parallel mixed-methods survey design was employed, collecting quantitative and qualitative data through a SurveyMonkey survey.

### 2.2. Participants and Recruitment

Participants were recruited through flyers posted on social media platforms, including X, Facebook, Reddit, and LinkedIn. Participants were offered entry into a voluntary raffle for one of three $50 Amazon gift cards.

**Figure 1:**
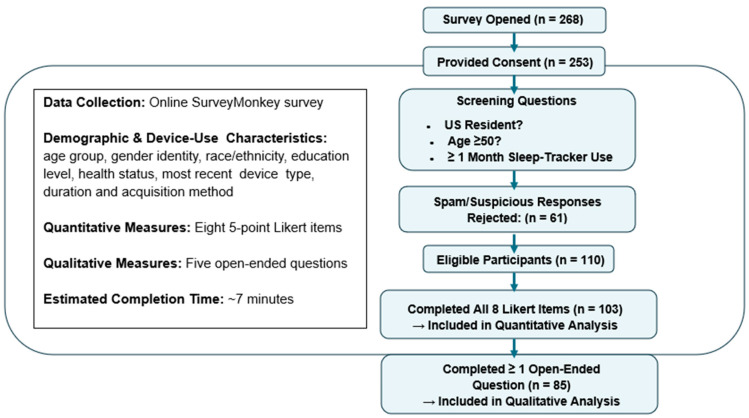
Flow diagram of participant inclusion.

Eligible participants were those who (1) provided informed consent, (2) were aged 50 years or older, (3) resided in the United States, and (4) reported current or prior use of a consumer sleep-tracking wearable device for at least one month. A total of *n* = 268 survey responses were recorded. Survey responses were screened for eligibility, completeness, and response quality. Entries identified as spam (*n* = 3) or exhibiting response patterns suggestive of automated or otherwise suspicious submissions (*n* = 58) were excluded prior to analysis. For a detailed overview of the participant flow, screening process, and final analytic samples, see Figure 1.

After applying eligibility criteria and excluding incomplete or suspicious responses, 110 participants remained in the eligible sample. Of these, seven otherwise eligible participants were excluded from quantitative analyses due to incomplete responses to one or more Likert-scale items required for composite score calculation. A total of 103 participants who completed all eight Likert-scale items were included in the quantitative analytic sample, and 86 participants who provided at least one interpretable response to an open-text survey question were included in the qualitative analysis.

### 2.3. Survey Design

The survey included demographic questions (age group, gender identity, race/ethnicity, education level, and self-rated health), device-use characteristics (most recent device type, duration of use, and method of acquisition), eight Likert-scale items assessing perceived usefulness and behavioral influence, and five open-text questions examining bedtime and morning routines. Open-text questions explored bedtime and morning routines in greater depth, whereas Likert-scale items focused primarily on perceived usability, usefulness, and behavioral influence. These constructs were included to contextualize how engagement with sleep-tracking data may shape perceived behavioral influence and ongoing device use. The estimated survey completion time was seven minutes. The full survey instrument is presented in Appendix A.

### 2.4. Survey Pilot Testing

The investigator-developed survey was pilot tested with six individuals familiar with sleep-tracking wearables to ensure clarity, usability, and appropriate length.

### 2.5. Statistical Analysis

Likert-scale responses were coded from 1 (Strongly Disagree) to 5 (Strongly Agree). Descriptive statistics included means (SD) and medians (IQR). Response distributions were additionally summarized using top-box (Agree/Strongly Agree), neutral, and bottom-box (Strongly Disagree/Disagree) percentages.

To test the primary hypothesis, a behavior-change composite score was calculated as the mean of two pre-specified Likert-scale items: (1) *“Using my sleep-tracking data has led to positive changes in my bedtime routine”* and (2) *“Sleep-tracking devices support long-term improvements in my sleep habits.”* Only the behavior-change items were combined into a composite, as these items directly operationalized the primary hypothesis concerning perceived behavioral influence. Other Likert-scale items were analyzed individually to preserve conceptual distinctions among usability, perceived usefulness, and emotional response constructs. The composite score was evaluated against the neutral midpoint value of 3 using a one-sample Wilcoxon signed-rank test. This nonparametric approach was selected due to the ordinal scaling of Likert responses and to avoid assumptions of normality. For secondary aims, associations between the behavior-change composite and demographic or contextual variables (gender, age group, education level, self-rated health status, most recent device type, duration, and method of acquisition) were examined using nonparametric group comparison tests. Mann–Whitney U tests were used for two-group comparisons (gender), and Kruskal–Wallis tests were used for variables with three or more categories. Categories with small cell sizes were collapsed into an “Other” grouping to improve statistical stability. All subgroup analyses were interpreted as exploratory.

Open-text responses were analyzed using reflexive thematic analysis as described by Braun and Clarke (2006). Responses were reviewed iteratively and coded inductively within an Excel spreadsheet. Consistent with reflexive thematic analysis (Braun & Clarke, 2006), coding and theme development were conducted by the first author rather than through inter-rater reliability procedures. Themes were developed inductively through iterative review of responses, comparison of coded excerpts, and refinement of conceptual groupings. Each response segment was assigned to one or more developing themes using a color-coded system to support visual organization and pattern identification. Themes were refined through repeated review to ensure internal consistency and clear distinctions between categories.

Quantitative and qualitative findings were integrated using joint displays to examine convergence, complementarity, and divergence across data sources. Integration focused on explaining how and why perceived behavioral influence varied across participants and contextual factors, consistent with the convergent parallel mixed-methods framework.

## 3. Results

Quantitative and qualitative results were analyzed independently and later synthesized to develop integrated interpretations.

### 3.1. Participant Characteristics

The 110 eligible responses were used to summarize participant demographics and device-use characteristics. Participants were primarily aged 50–59 years (45.5%), followed by 60–69 (32.7%) and 70–79 (20.0%). The sample was predominantly female (72.7%) and largely White (80.9%), with smaller proportions identifying as Hispanic or Latino (6.4%), Black or African American (5.5%), multiracial (3.6%), or other racial or ethnic backgrounds (3.6%).

Participants were generally well educated, with the majority reporting completion of a graduate degree (42.7%) or bachelor’s degree (33.6%), followed by associate degrees (10.0%) and some college education (9.1%). Most respondents reported their overall health as very good (46.4%) or excellent (21.8%), with fewer reporting good (25.5%) or fair/poor (5.5%).

Regarding device use, the most commonly reported sleep-tracking device was the Apple Watch (44.5%), followed by Oura Ring (21.8%), Fitbit (10.0%), WHOOP (9.1%), and Samsung Galaxy (4.6%). Most participants reported long-term use, with 57.3% using their device for more than one year. Most devices were self-purchased (77.3%), while 18.2% were received as gifts, and a small proportion were provided through wellness programs or for medical reasons. Full demographic and device-use characteristics are presented in Appendix B Table A1.

### 3.2. Quantitative Findings

Participants (*n* = 103) reported generally favorable perceptions of sleep-tracking wearables across measures of usability, usefulness, and behavioral influence. Ease of use and understanding received high ratings (M = 4.14, SD = 0.86), with 84.5% of respondents endorsing top-box responses (agree or strongly agree). Similarly, perceived usefulness of sleep data demonstrated strong agreement (M = 4.27, SD = 0.72), top-box = 89.3%. A large majority also agreed that the benefits of sleep-tracking devices outweigh associated frustrations (M = 4.19, SD = 0.82, top-box = 83.5%), and 75.7% reported that sleep trackers encouraged healthier sleep habits (M = 4.04, SD = 0.93).

In contrast, tracking-related stress or anxiety was relatively low (M = 2.62, SD = 1.11), with more than half of respondents (54.4%) endorsing bottom-box responses (disagree or strongly disagree). Descriptive statistics for Likert-scale survey items are presented in Table 1.

The behavior-change composite was 3.78 (SD = 0.87), with a median of 4 (IQR = 1.5). A one-sample Wilcoxon signed-rank test indicated that scores were significantly greater than the neutral midpoint (V = 3578, *p* < 0.001), with a large effect size (r = 0.67).

Exploratory subgroup analyses demonstrated a small but statistically significant association between gender and behavior-change composite scores (U = 712, *p* = 0.024, r = 0.22), with female participants demonstrating slightly more favorable score distributions overall despite similar median scores across groups. Self-rated health status was also significantly associated with composite scores (H(3) = 7.92, *p* = 0.048, η^2^ = 0.050). Participants reporting fair or poor health demonstrated lower composite scores than those reporting excellent, very good, or good health. No statistically significant differences were observed across age group, education level, race/ethnicity, device type, duration of device use, or device acquisition method (all *p* > 0.05). Detailed hypothesis testing and exploratory results are presented in Appendix B, Table A2 and Table A3.

### 3.3. Qualitative Themes (Themes and Codes)

Reflexive thematic analysis of open-text responses (*n* = 86) identified four primary themes describing how older adults experience, interpret, and incorporate sleep-tracking data into their bedtime and morning routines.

#### 3.3.1. Theme 1: Sleep Tracking as a Reflective Daily Feedback System (*n* = 30)

Many participants described reviewing sleep data as part of a routine morning “check-in,” using device metrics to reflect on the previous night’s sleep and inform decisions. Sleep-tracking data were commonly framed as a feedback tool that increased awareness of sleep patterns over time, rather than as prescriptive instructions. Codes associated with this theme included morning check-ins, reviewing sleep scores, trend monitoring, linking sleep metrics to daytime readiness, and reflective self-monitoring.

Participants frequently described starting their day by checking their sleep score or reviewing sleep summaries. One participant noted, *“I check the sleep score every morning and find it interesting and a reminder that going to bed earlier definitely makes a difference.”* Others described using the data to make sense of how rested or tired they feel upon waking, or to inform daily activities: *“A ‘sleep score’ is helpful when trying to assess how hard my daily workout should be.”* Across responses, sleep-tracking data were most often positioned as informational feedback that supported reflection and self-awareness.

#### 3.3.2. Theme 2: Incremental Adjustment of Bedtime Routines (*n* = 46)

Over half of respondents described making small adjustments to their bedtime routines based on insights from sleep-tracking data. These changes were typically framed as minor routine modifications rather than major lifestyle transformations. Codes contributing to this theme included earlier bedtimes, wind-down routine changes, reduced evening screen use, adjustments to caffeine or alcohol consumption, bedtime reminders, and minor routine tweaks.

Participants frequently described subtle behavioral shifts in their evening habits. One participant reported, *“I try to put my phone down sooner because I can see it affects my sleep score.”* Others described how sleep-tracking data encouraged more consistent sleep hygiene behaviors. As one respondent shared, *“It nudges me to make better choices at night.”* These responses suggest that participants often viewed sleep-tracking devices as behavioral prompts that encouraged gradual improvements in bedtime routines over time.

#### 3.3.3. Theme 3: Selective Interpretation and Conditional Trust in Metrics (*n* = 35)

Many participants described interpreting sleep-tracking metrics selectively, weighing device outputs against their own subjective sleep experiences. Codes associated with this theme included accuracy skepticism, doubts about sleep-stage and sleep quality data, comparing device output to subjective experience, treating irregular nights as outliers, and selectively using certain metrics while disregarding others. Participants frequently described evaluating the data critically. One participant commented, “*Sometimes the data doesn’t match how I actually feel. For example, it might say I had ‘great’ sleep when I feel tired, or show wake-ups that I don’t remember.*” Similarly, another participant claimed, “*I use it as a guide, not gospel.*” These responses suggest that participants often integrated device feedback with personal experience, applying conditional trust to sleep-tracking metrics rather than accepting them at face value.

#### 3.3.4. Theme 4: Design Friction and Desire for More Actionable Guidance (*n* = 32)

Participants also identified usability constraints and limitations in device feedback, often expressing a desire for clearer, more actionable sleep guidance. Contributing codes included charging difficulties, comfort or fit issues, alerts interfering with sleep continuity, app or connectivity problems, and a desire for more tailored recommendations or actionable coaching.

Several respondents expressed frustration with the limited behavioral guidance provided by their devices. One participant remarked, *“I would like my sleep tracker to give more personalized and practical insights instead of just showing raw data.”* Others highlighted physical usability concerns, such as difficulty sleeping with the device, with one individual observing, *“It is very difficult to sleep with a device on my arm.”* Together, these responses highlight participants’ interest in more personalized, actionable sleep guidance that better translates sleep-tracking data into practical behavioral recommendations.

#### 3.3.5. Thematic Synthesis

Taken together, these themes indicate that sleep-tracking devices primarily support awareness and reflection of sleep patterns, rather than driving substantial behavioral change. At the same time, respondents noted their usefulness depended on how easily the data could be interpreted and whether devices provided practical guidance. A detailed thematic analysis summarizing themes, codes, and representative quotes is shown in Appendix B, Table A4.

### 3.4. Integrated Findings

Joint display analysis demonstrated complementarity between quantitative ratings of usability and qualitative descriptions of reflective engagement with sleep-tracking data. Quantitative results indicate strong agreement regarding device usability and usefulness, including ease of use, usefulness of sleep data, and benefits outweighing frustrations. These findings aligned with qualitative accounts describing sleep tracking as a reflective feedback system in which participants reviewed sleep scores, monitored sleep trends over time, and used the data to interpret sleep and inform daily activities (30 respondents; 34.9%). Together, these results suggest that users generally perceived sleep-tracking wearables as accessible tools that support awareness and interpretation of sleep patterns.

Moderate quantitative ratings related to behavioral influence converged with qualitative descriptions of incremental routine adjustment. Quantitative findings indicated behavior-change scores above the neutral midpoint, suggesting a positive influence on sleep-related behaviors. Qualitative findings supported this, with over half of respondents describing modest adjustments such as earlier bedtimes, reduced late-night screen use, or improved wind-down routines. These complementary findings suggest sleep-tracking devices may encourage behavioral change, but these changes are typically gradual rather than transformative.

Quantitative perceptions of device reliability and usefulness also complemented qualitative reports of selective interpretation. Although perceived accuracy and reliability were rated relatively high (M = 3.93; top-box = 78.6%), many respondents described evaluating device outputs with their own subjective experiences (35 respondents; 40.7%). Participants often compared metrics to perceived restfulness, questioned sleep-stage data, and dismissed unusual nights as outliers. These findings suggest that users generally value sleep-tracking data while simultaneously applying conditional trust when interpreting device metrics.

Finally, quantitative findings indicated low levels of stress or anxiety related to sleep tracking, which contrasted slightly with qualitative expressions of usability friction and a desire for actionable guidance. Tracking-related stress and anxiety received low agreement (M = 2.62; bottom-box = 54.4%), indicating that most participants did not experience significant negative emotional responses to sleep monitoring. However, qualitative responses identified several limitations, including device comfort issues, charging requirements, and limited actionable guidance (32 respondents; 37.2%). Participants frequently expressed interest in personalized sleep recommendations that would translate device metrics into practical strategies.

Taken together, the integrated findings suggest that sleep-tracking wearables function primarily as tools that increase awareness of sleep patterns and support reflective engagement with bedtime and morning routines. While these devices appear capable of encouraging modest behavioral adjustments, their perceived usefulness is moderated by how clearly the data can be interpreted and whether devices provide actionable sleep guidance. An integrated joint display of quantitative findings and qualitative themes is presented in Appendix B, Table A5.

## 4. Discussion

### 4.1. Sleep Awareness

Rather than directly modifying sleep behavior, the findings from this study suggest sleep-tracking wearables operate largely as awareness technologies that help older adults observe and reflect on habits influencing sleep. Participants reported high satisfaction, with more than 80% endorsing ease of use and perceived benefit. Qualitative responses described how device feedback increased awareness of sleep patterns and daily routines. These findings align with prior research indicating that personal health monitoring often influences behavior indirectly by increasing engagement with health data rather than prescribing change (Del-Valle-Soto et al., 2024; Hoffmann & Gualtieri, 2026). In this context, wearable devices may translate abstract concepts such as “sleep hygiene” into observable patterns that users can interpret within everyday routines.

### 4.2. Digital Engagement

An additional insight emerging from this study is that adults aged 50 years and older appear far more comfortable with wearable technologies than is often assumed. Participants reported high usability and low frustration across multiple device types and durations of use, challenging persistent stereotypes that older adults uniformly struggle to engage with digital health technologies. That said, these findings should be interpreted in the context of the sample characteristics, as nearly half of the participants were between 50 and 59 years of age, and the sample was predominantly highly educated and technologically engaged.

These observations are consistent with a growing body of research demonstrating that older adults are increasingly active participants in digital health ecosystems rather than passive observers of technological change (Ghazi et al., 2025). National survey data similarly show that many adults aged 50 years and older regularly use smartphones, smart devices, and health-tracking applications to support independence and health management (Kakulla, 2024; Kakulla & AARP Research, n.d.). As consumer health technologies continue to expand, older adults may represent an increasingly engaged population using digital tools to better understand and manage their health behaviors.

### 4.3. Incremental Habit Change

Although quantitative analyses showed that behavior-change composite scores were significantly greater than the neutral midpoint, qualitative findings suggest that the influence of sleep-tracking devices was generally incremental rather than transformative. Participants described modest adjustments consistent with sleep hygiene practices, including earlier bedtimes, reduced evening screen use, and more regular sleep schedules. This pattern is consistent with prior survey research showing that middle-aged and older adults already attempt to improve sleep through strategies such as maintaining consistent bedtimes, limiting late-day caffeine intake, and optimizing the sleep environment (Lampkin, 2024b).

These gradual behavioral adjustments can be interpreted through the Transtheoretical Model of behavior change (Kononova et al., 2019; Hui & Grandner, 2015; Prochaska & Velicer, 1997). Sleep-tracking devices appear to support contemplation and preparation by increasing awareness, reinforcing self-monitoring, and strengthening behavioral consistency rather than precipitating rapid transformation. Within this framework, micro-changes represent iterative refinements as individuals move toward action and maintenance. However, progression toward later stages of change is not automatic.

### 4.4. Exploratory Demographic Associations

Perceived behavioral influence appeared to vary modestly across participant characteristics. Female participants demonstrated slightly more favorable behavior-change score distributions, while participants reporting fair or poor health demonstrated lower composite scores than those reporting better overall health. This finding may reflect the broader interaction between sleep, health status, and daily functioning observed in older adults (Li et al., 2018). Because subgroup sizes were unequal and analyses were exploratory, these findings should be interpreted cautiously and viewed as hypothesis-generating rather than confirmatory.

### 4.5. Interpreting Sleep Data

Participants in this study did not treat wearable sleep metrics as definitive indicators of sleep quality. Instead, many interpreted device feedback alongside their own experiences of restfulness and daily functioning. This pattern suggests that users maintain autonomy when evaluating wearable sleep data rather than fully deferring to algorithmic output. Prior research similarly shows that subjective perceptions of sleep quality often differ from objectively measured sleep parameters, particularly in older adults (Li et al., 2018). Age-related changes in sleep architecture and the tendency for consumer devices to misclassify motionless wakefulness as sleep may contribute to these discrepancies (Willoughby et al., 2024). These limitations highlight the need to interpret wearable sleep metrics within the broader context of user experience.

### 4.6. Digital Companions

Overall, the findings of this study suggest that consumer sleep-tracking wearables may be best understood not as direct behavioral interventions but as reflective digital companions that support awareness and gradual habit formation. By allowing users to visualize sleep patterns and connect nightly behaviors with daytime experiences, these technologies may encourage ongoing engagement with sleep health. In clinical settings, wearable sleep data may therefore be most valuable as a conversation starter, helping individuals and healthcare providers explore how daily habits influence sleep and daytime functioning.

## 5. Limitations

Despite its contributions, this study has limitations that should be considered when interpreting the findings. The study relied on self-reported perceptions of behavioral change rather than objectively measured sleep or behavioral outcomes. Although perceived influence is central to understanding lived experience and user engagement, reported improvements may not directly correspond to measurable behavioral outcomes. Additionally, the cross-sectional design limits causal interpretation, making it difficult to determine whether sleep tracking influenced behavior or whether individuals already motivated to improve sleep were more likely to adopt and engage with these devices.

Recruitment through publicly accessible social media platforms may have introduced sampling bias toward individuals who are technologically engaged and comfortable with digital tools. This is reflected in the sample characteristics, which were predominantly White, female, and highly educated. In addition, some subgroup categories contained small sample sizes and were therefore collapsed to improve analytic stability. Accordingly, subgroup findings should be interpreted cautiously.

Exploratory subgroup analyses identified a modest association between gender and behavior-change composite scores; however, this finding should be interpreted cautiously given the unequal subgroup sizes. Individuals who discontinued device use due to dissatisfaction, technical barriers, or negative experiences may be underrepresented, potentially inflating perceived benefit estimates. Collectively, these factors may limit generalizability to more socioeconomically and racially diverse populations and to older adults with lower digital literacy.

Because the survey link was publicly accessible, there was potential risk for automated or fraudulent responses. To mitigate this risk, responses were systematically screened for duplicate entries, implausibly short completion times, inconsistent eligibility responses, and patterned or non-meaningful answering. A total of 61 entries were excluded as suspicious, incomplete, or likely spam. While screening procedures substantially reduced the likelihood of fraudulent data, the possibility of undetected automated responses cannot be excluded.

The survey instrument was investigator-developed and not previously validated; therefore, formal psychometric properties were not established. Although Likert-scale responses were analyzed using appropriate nonparametric methods, ordinal self-report measures remain subject to response bias and social desirability. In addition, participants used multiple consumer-grade devices with differing proprietary algorithms and feedback mechanisms, limiting attribution of behavioral perceptions to any specific platform.

Finally, qualitative analysis relied on written open-text responses rather than in-depth interviews, which may have limited contextual depth. However, integrating quantitative and qualitative findings strengthened interpretive credibility through methodological triangulation.

## 6. Conclusions

Among adults aged 50 years and older, consumer sleep-tracking wearables are perceived as accessible and useful tools that support positive sleep-related behaviors. Quantitative findings indicate behavior change above neutrality, while qualitative findings suggest these changes are typically gradual rather than transformative. Rather than driving dramatic shifts, these devices facilitate small adjustments that increase awareness and refine sleep routines. By preserving autonomy and supporting sustained self-management, sleep-tracking technologies may help make “aging in place” a more achievable reality.

Future research should examine longitudinal behavioral outcomes to determine whether perceived micro-changes translate into sustained physiological or sleep-related improvements. Establishing whether incremental adjustments accumulate into measurable changes would strengthen causal interpretation.

Additionally, a mixed-methods study comparing clinician interpretations of consumer sleep-tracking data with the perceptions reported in this survey could clarify areas of alignment and divergence. Understanding concordance (or discrepancy) between patient and clinician perspectives may inform responsible integration of wearable data into sleep medicine practice.

Finally, future work should explore the cognitive effects of sleep feedback, particularly given that a subset of participants in this study endorsed tracking-related stress or anxiety. Specifically, does viewing a low sleep score alter perceived sleepiness, fatigue, or mood independent of objective sleep? Investigating potential expectancy or nocebo effects would clarify whether wearable summaries shape not only behavior, but also subjective experience.

## Figures and Tables

**Table 1:** Descriptive statistics for Likert-scale survey items.

Item	N	M (SD)	Median (IQR)	Top-Box%	Neutral %	Bottom-Box %
Ease of use/understand	103	4.14 (0.86)	4 (1)	84.5	10.7	4.9
Perceived accuracy/reliability	103	3.93 (0.78)	4 (0)	78.6	16.5	4.9
Usefulness of sleep data	103	4.27 (0.72)	4 (1)	89.3	9.7	1.0
Benefits outweigh frustrations	103	4.19 (0.82)	4 (1)	83.5	13.6	2.9
Encourages healthier habits	103	4.04 (0.93)	4 (1)	75.7	17.5	6.8
Stress/anxiety from tracking	103	2.62 (1.11)	2 (2)	27.1	18.5	54.4
Positive bedtime routine change	103	3.70 (1.03)	4 (1)	63.1	22.3	14.6
Long-term sleep habit improvement	103	3.85 (0.83)	4 (1)	72.8	22.3	4.9

Note. M = mean; SD = standard deviation; IQR = interquartile range. Top-box represents the percentage of respondents selecting “Agree” or “Strongly Agree”, neutral represents “Neutral,” and bottom-box represents “Disagree” or “Strongly Disagree”.

## Data Availability

The raw data supporting the conclusions of this article will be made available by the authors upon reasonable request. Data are not publicly available due to ethical and privacy restrictions related to participant confidentiality and the terms of informed consent.

## Appendix A. IRB-Approved Survey Instrument

Note: The following pages reproduce the IRB-approved SurveyMonkey questionnaire used to collect study data. Skip logic and page breaks are included to reflect the original survey structure.

**Sleep Tracking Survey** **Survey Flow**
**Q1: Consent Question (Required):**
*Do you consent to participate in this study?* - Yes, I agree - No
**Skip Logic:** If “No” → If you would like to enter the raffle to win 1 of 3 $50 Amazon gift cards, please select ‘Yes’ to the question below, and you will be provided with a link to a separate survey where you can enter your email address. **This step is optional.** The raffle will be one month after the survey closes.

If you would not like to participate, please select ‘No’ below.

**Q29: Would you like to participate in the raffle?**
- Yes - No

*Skip Logic:* If “No” → End Survey If “Yes”—Link to survey “Emails for Raffle” Survey preview
**SCREENING**
**Q2: Are you currently in the United States? (Required)** - Yes - No
*Skip Logic:* If “No” → End Survey
**Q3: Are you 50 years old or older? (Required)**
- Yes - No
*Skip Logic:* If “No” → End Survey
**Q4: Have you ever worn a sleep-tracking device (Fitbit, Apple Watch, Oura, etc.) consistently for at least 1 month? *(Required)***
- Yes - No
**Skip Logic:**
- If “No” → End Survey - If “Yes” → Show Q5
**Q5: Which sleep-tracking device(s) have you used?** *Select all that apply.*
- Fitbit - Apple Watch - Oura Ring - Garmin - WHOOP - Samsung Galaxy Watch - Prefer not to say - Other: ________
**Q6: Which sleep-tracking device have you used most recently?**
- Fitbit - Apple Watch - Oura Ring - Garmin - WHOOP - Samsung Galaxy Watch - Prefer not to say - Other: ________
**Q7: How long have you used (or did you use) your most recent sleep-tracking device?**
- Less than 1 month - 1–3 months - 4–6 months - 7–12 months - More than 1 year - Prefer not to say
**Q8: How did you get your most recent sleep-tracking device?**
- I purchased it myself - It was gifted to me - It was provided through a wellness program or employer - It was provided for medical/health reasons - Prefer not to say - Other:
**Q9: Do you use your sleep-tracking device for reasons other than tracking your sleep? For example, do you use it for tracking activity tracking, or heart-rate monitoring?**
- Yes - No - Not sure - Prefer not to say
**If “Yes,” ask:**
**Q10: What other reasons do you use your device for?**
***Select all that apply.***
- Activity tracking/steps - Heart-rate monitoring - Exercise/workout tracking - Stress monitoring - Blood oxygen (SpO_2_) monitoring - Notifications or communication - Prefer not to say - Other: ________
**Demographics (5 Items)**
**Q11: What is your age?**
- 50–59 - 60–69 - 70–79 - 80–89 - 90+ - Prefer not to say
**Q12: What is your gender?**
- Male - Female - Prefer not to say
**Q13: What is your race and/or ethnicity? (Select all that apply)**
[Checkboxes]
- Asian - Black or African American - Hispanic or Latino - Native American or Alaska Native - Native Hawaiian or Pacific Islander - White - Prefer not to say - Other: ______
**Q14: What is the highest level of education you have completed?**
- Less than high school - High school diploma/GED - Some college - Associate degree - Bachelor’s degree - Graduate or professional degree - Prefer not to say
**Q15: How would you rate your overall health?**
- Excellent - Very good - Good - Fair - Poor - Prefer not to say
**LIKERT SCALE QUESTIONS (8 Items)**
**Instructions:**
*Please choose how much you agree with the following statements.* *Scale: 1 = Strongly Disagree, 2 = Disagree, 3 = Neutral, 4 = Agree, 5 = Strongly Agree.*
**Theme 1: Experience & Usability**
**Q16: Sleep-tracking devices are easy for older adults (aged 50+) to use and understand.**
[1–5 Likert Scale]

**Q17: The data about sleep that my device shows is accurate and reliable.**
[1–5 Likert Scale]
**Theme 2: Perceived Usefulness**
**Q18: My most recent sleep-tracking device provides (or has provided) useful information that helps me understand my sleep habits.**
[1–5 Likert Scale]

**Q19: The benefits of using my sleep tracker outweigh any frustrations or challenges.**
[1–5 Likert Scale]
**Theme 3: Sleep Hygiene Practices**
**Q20: My sleep tracker encourages healthier sleep habits (e.g., consistent bedtimes, reduced screen use).**
[1–5 Likert Scale]

**Q21: Tracking my sleep data sometimes causes stress or anxiety.** *(Reverse-score if needed)*
[1–5 Likert Scale]
**Theme 4: Behavior Change & Outcomes**
**Q22: Using my sleep-tracking data has led to positive changes in my bedtime routine.**
[1–5 Likert Scale]

**Q23: Sleep-tracking devices support long-term improvements in my sleep habits.**
[1–5 Likert Scale]
**OPEN-ENDED QUESTIONS**
**Q24: How has your sleep-tracking device affected your evening routine, or how you prepare for bedtime?**
(Open-ended)

**Q25: How has your sleep-tracking device affected your morning routine or how you start the day?**
(Open-ended)

**Q26: What parts of your sleep tracker do you find most helpful or useful?**
(Open-ended)

**Q27: What challenges or frustrations have you had while using a sleep-tracking device?**
(Open-ended)

**Q28: What would you like your sleep tracker to do differently that would make it more useful to you?**
(Open-ended)

**THANK YOU PAGE**

**Title:** *Thank You!*

**Text:**
Thank you for completing this survey. Your responses have been recorded. If you would like to enter the raffle to win 1 of 3 $50 Amazon gift cards, please select ‘Yes’ to the question below, and you will be provided with a link to a separate survey where you can enter your email address. **This step is optional**. Your email **cannot be linked** to your survey responses. The raffle will be one month after the survey closes.

If you would not like to participate, please select ‘No’ below.

**Q29: Would you like to participate in the raffle?**
- Yes - No
*Skip Logic:*
If “No” → End Survey
If “Yes”—Link to survey “Emails for Raffle” Survey preview

Thank you for completing this survey. Please select the link “Emails for Raffle” for raffle entry.

## Appendix B

**Table A1:** Demographics and Device-Use Characteristics.

Age group	*n*	%	(%)
50–59	50	45.5	
60–69	36	32.7	
70–79	22	20.0	
80–89/Other	2	1.80	
Gender	*n*	%	(%)
Female	80	72.73	
Male	30	27.27	
Race/ethnicity	*n*	%	(%)
White	89	80.91	
Hispanic or Latino	7	6.36	
Black or African American	6	5.45	
Multiracial	4	3.64	
Other	4	3.64	
Education Level	*n*	%	(%)
Graduate degree	47	42.72	
Bachelor’s degree	37	33.64	
Associate degree	11	10.00	
Some college	10	9.09	
High school diploma/GED	4	3.64	
Prefer not to say	1	0.91	
Self-rated health status	*n*	%	(%)
Excellent	24	21.82	
Very good	51	46.36	
Good	28	25.45	
Fair	5	4.55	
Poor	1	0.91	
Prefer not to say	1	0.91	
Most recent device type	*n*	%	(%)
Apple Watch	49	44.54	
Oura Ring	24	21.82	
Fitbit	11	10.00	
WHOOP	10	9.09	
Samsung Galaxy Watch	5	4.55	
Garmin	3	2.73	
Other	6	7.27	
Duration of use	*n*	%	(%)
More than 1 year	63	57.27	
7–12 months	11	10	
4–6 months	16	14.55	
1–3 months	13	11.82	
Less than 1 month	7	6.36	
Acquisition method	*n*	%	(%)
Purchased myself	85	77.27	
Gifted to me	20	18.18	
Wellness program/employer	4	3.64	
Medical/health reasons	1	0.91	

Note. Values represent the number and percentage of participants within each category (*N* = 110). Percentages may not sum to exactly 100% due to rounding. Device-use characteristics refer to the participant’s most recently used sleep-tracking device.

**Table A2:** Quantitative Analysis. Primary hypothesis test: Behavior-Change Composite vs neutral midpoint (*n* = 103).

Outcome	Test	Mean (SD)	Median (IQR)	Neutral Reference	Test Statistic	*p*	Effect Size (r)
Behavior-change composite (mean of bedtime routine change + long-term improvement)	One-sample Wilcoxon signed-rank	3.78 (0.87)	4 (1.50)	3	V = 3578	<0.001	0.67

Note. The Behavior-Change Composite is the mean of two Likert items assessing bedtime routine change and long-term sleep improvement.

**Table A3:** Quantitative Analysis. Secondary hypothesis test: Exploratory associations with the Behavior-Change Composite (*n* = 103).

Predictor	Test	Group Medians (IQR)	Test Statistic	*p*	Effect
Gender	Mann–Whitney U	Female: 4 (1.00), Male: 4 (1.25)	U = 712	**0.024**	r = 0.223
Age group	Kruskal–Wallis	50–59: 4 (0.50), 0–69: 4 (1.63), 70–79: 4 (1.00), Other: 3 (0.00)	H(3) = 3.38	0.336	η^2^ = 0.004
Education	Kruskal–Wallis	High School: 3 (0.25), Some college: 4 (0.50), Associate: 4 (0.50), Bachelor’s: 4 (1.50), Graduate: 4 (1.00)	H(4) = 3.66	0.455	η^2^ < 0.001
Self-rated health	Kruskal–Wallis	Excellent: 4 (1.38), Very Good: 4 (0.50), Good: 4 (1.00), Fair/Poor: 3.25 (0.88)	H(3) = 7.92	**0.048**	η^2^ = 0.050
Race/ethnicity	Kruskal–Wallis	Black/AA: 4.25 (1.25), Hispanic/Latino: 4 (0.38), Multiracial: 3.5 (1.50), White: 4 (1.00), Other: 4.25 (1.25)	H(4) = 1.42	0.840	η^2^ < 0.001
Device type	Kruskal–Wallis	Apple Watch: 4.0 (0.50), Fitbit: 3.75 (2.00), Garmin: 3.5 (1.25), Oura Ring: 4(1.25), Samsung Galaxy: 2.75 (1.13),Other: 4 (0.375)	H(5) = 4.80	0.441	η^2^ < 0.001
Duration of use	Kruskal–Wallis	<1 Month: 3.50 (0.50), 1–3 Months: 3.5 (0.50), 4–6 Months: 3.75 (0.50) 7–12 Months: 4 (0.75), >1 Year: 4 (1.00)	H(4) = 2.72	0.606	η^2^ < 0.001
Device acquisition	Kruskal–Wallis	Purchased: 4 (1.125), Gifted: 4 (1.00), Other: 4 (0.50)	H(2) = 0.38	0.825	η^2^ < 0.001

Note. Values represent group medians (IQR). Mann–Whitney U tests were used for two-group comparisons and Kruskal–Wallis tests for multi-group comparisons. Bold *p*-values indicate statistical significance (*p* < 0.05). Effect sizes are reported as r (Mann–Whitney U) and η^2^ (Kruskal–Wallis).

**Table A4:** Thematic analysis of open-text survey responses.

Theme	Definition	Codes	*n* (%) of Respondents	Representative Quote(s)
Sleep Tracking as a Reflective Daily Feedback System	Participants described using sleep metrics as a routine, reflective check-in, typically conducted in the morning, allowing them to interpret data from previous night and increase awareness of sleep patterns over time.	Morning check-in, Reviewing sleep scores, Trend monitoring, Linking data to daytime readiness, Reflective self-monitoring	30 (34.9%)	“I check the sleep score every morning and find it interesting and a reminder that going to bed earlier definitely makes a difference.” “A ‘sleep score’ is helpful when trying to assess how hard my daily workout should be.”
Incremental Adjustment of Bedtime Routines	Participants reported modest, practical adjustments to their bedtime routines, framed as small tweaks rather than major or sustained lifestyle change.	Earlier bedtime, Wind-down changes, Reduced screen use, Caffeine/alcohol reduction, Bedtime reminders, Minor routine tweaks	46 (53.5%)	“I try to put my phone down sooner because I can see it affects my sleep score.” “It nudges me to make better choices at night.”
Selective Interpretation and Conditional Trust in Metrics	Participants critically evaluated tracker outputs. Conditional trust was granted after weighing subjective sleep experiences alongside device metrics and discounting results that appeared discordant.	Accuracy skepticism, Sleep stage/quality doubt, Comparing metrics to subjective experience, Treating single-night irregularities as outliers, Selective metric use	35 (40.7%)	“Sometimes the data doesn’t match how I actually feel. For example, it might say I had “great” sleep when I feel tired, or show multiple wake-ups that I don’t remember. That can make me question how reliable the metrics are.” “I use it as a guide, not gospel.”
Design Friction and Desire for More Actionable Guidance	Participants identified usability constraints and practical burdens, expressing a preference for more personalized and actionable recommendations rather than isolated scores or raw data.	Charging difficulties, Comfort/fit issues, Alerts interfering with sleep continuity, App/connectivity problems, Desire for tailored recommendations, Actionable coaching	32 (37.2%)	“I would like my sleep tracker to give more personalized and practical insights instead of just showing raw data.” “It is very difficult to sleep with a device on my arm.”

Note. Thematic analysis was conducted on open-text survey responses provided by eligible participants (*n* = 86). Percentages represent the proportion of respondents whose comments reflected each theme. Responses could be coded to multiple themes. Quotes are illustrative examples.

**Table A5:** Joint Display of Quantitative Findings and Qualitative Themes.

Theme	Quantitative Evidence (*n* = 103)	Qualitative Evidence (*n* = 86)	Integration	Meta-Inference
Sleep Tracking as a Reflective Daily Feedback System	Usefulness of sleep data: *M = 4.27, Top-box = 89.3%* Ease of use: *M = 4.14, Top-box = 84.5%* Benefits outweigh frustrations: *M = 4.19, Top-box = 83.5%*	Morning review of sleep scores and pattern awareness. (*n* = 30; 34.9%)	**Complementary**	Sleep tracking primarily functions as a reflective awareness tool supporting interpretation of sleep patterns and daily readiness.
Incremental Adjustment of Bedtime Routines	Positive bedtime routine change: *M = 3.70, Top-box = 63.1%* Long-term improvement: *M = 3.85, Top-box = 72.8%* Behavior-change composite: *Median = 4; Wilcoxon p < 0.001, r = 0.67.*	Small routine tweaks such as earlier bedtime, reduced screen use, or improved wind-down routines. (*n* = 46; 53.5%).	**Convergent/** **Complementary**	Quantitative results confirm behavior-change scores significantly above neutral, while qualitative data show these adjustments were typically incremental.
Selective Interpretation and Conditional Trust in Metrics	Perceived accuracy/reliability: *M = 3.93, Top-box = 78.6%* Usefulness of sleep data: *M* = *4.27, Top-box = 89.3%.*	Participants compared device outputs with subjective sleep experience and discounted metrics perceived as inaccurate. (*n* = 35; 40.7%).	**Complementary**	Users value sleep data but interpret device metrics selectively rather than accepting them uncritically.
Design Friction and Desire for More Actionable Guidance	Stress/anxiety from tracking: *M = 2.62, Top-box = 27.2%* Benefits outweigh frustrations: *M = 4.19, Top-box = 83.5%* Ease of use: *M = 4.14, Top-box = 84.5%.*	Participants reported charging issues, comfort concerns, and desire for personalized recommendations. (*n* = 32; 37.2%).	**Complementary**	Devices are widely accepted and useful but perceived as limited in actionable coaching or tailored guidance.

Note: Quantitative analyses were conducted on respondents who completed all eight Likert-scale items (*n* = 103). Qualitative themes were derived from respondents who answered at least one open-ended question (*n* = 86).
